# Chronically socially isolated mice exhibit depressive-like behavior regulated by the gut microbiota

**DOI:** 10.1016/j.heliyon.2024.e29791

**Published:** 2024-04-18

**Authors:** Linwei Ding, Jiaqi Liu, Yunjia Yang, Zeying Cui, Guankui Du

**Affiliations:** a Biotechnology Laboratory, Hainan Medical University, Haikou, China; b Biotechnology Major, Hainan Medical University, Haikou, China; c School of Public Health, Hainan Medical University, Haikou, China; d Department of Biochemistry and Molecular Biology, Hainan Medical University, Haikou, China; e Department of Breast Surgery, The First Affiliated Hospital of Hainan Medical University, Haikou, China

**Keywords:** Social isolation, Depression, Gut microbiota, Metabolite, Inflammatory responses

## Abstract

**Objectives:**

Chronic loneliness is a widespread issue, and the gut-brain axis is known to be crucial in facilitating communication between the gut and brain. However, the precise mechanism by which chronic loneliness affects the gut-brain axis remains uncertain.

**Methods:**

Fourteen 55-week-old Balb/c mice were used in the experiment, with seven mice being randomly assigned to the chronic social isolation (CSI) group. The CSI group mice underwent 12 weeks of isolation to simulate the psychiatric state of a population in prolonged social isolation. The mental state of the CSI mice was assessed through animal behavior analysis, while plasma cytokines were measured using ELISA. Additionally, the composition of the gut microbiota was analyzed using 16S rRNA sequencing, and the metabolite composition of the intestinal contents was examined using nontargeted metabolomics. The Student-T test was used to determine significant mean differences.

**Results:**

Mice that were exposed to the CSI exhibited increased immobility time lengths in forced swimming and hanging tail experiments, and decreased movement lengths and number of times traversing the intermediate region, compared to control mice. Additionally, CSI decreased the abundance of the probiotics Ruminococcaceae, Akkermansiaceae, and Christensenellaceae. Additionally, CSI reduced the production of the metabolites oleamide and tryptophan. Furthermore, IL-1β, IL-4, and IL-6 were significantly increased, while TNF-α was significantly decreased.

**Conclusion:**

CSI induces a dysbiotic gut microbiota and the production of neurorelated metabolites, which in turn increase inflammatory responses and result in depressive behaviors in CSI mice. Therefore, these findings suggest that the gut microbiota may serve as a target for the treatment of long-term social isolation-induced mental disorders.

## 1 Introduction

Social isolation refers to a state where an individual is completely or nearly isolated from society. In the context of the COVID-19 epidemic, individuals are socially isolated in specific physical spaces [1]. In addition, many elderly people are de facto socially isolated due to various reasons, including the death of their spouse, the absence of their children, and a limited social circle [2].

Social isolation can lead to feelings of loneliness and can undermine the sense of meaning in life [3]. This condition can result in serious mental health outcomes, such as depression [4]. Numerous studies have also indicated that individuals who experience social isolation are more likely to commit suicide than those who have strong social connections [5,6]. Conversely, studies have shown that being socially active may be an important factor in increasing life expectancy [7,8]. However, recent studies have revealed an increase in the number of people living alone between the ages of 45 and 65, which requires greater attention and vigilance from society [9,10].

Major depression can have a significant impact on both quality of life and physical health, and in severe cases, may even lead to suicide [11]. As individuals age and experience declining physical functioning, they may become more vulnerable to mood swings [10]. Depressive symptoms tend to be more severe in older adults [12]. A meta-analysis indicated that a high prevalence of depressive symptoms among elderly individuals in China was detected, while the prevalence of depression among the elderly in Guizhou and Hunan was as high as 50 % [13]. Furthermore, research has shown that the risk of mortality in older adults increases progressively with the severity of depressive symptoms, with those experiencing severe symptoms having a 2.27 times higher risk of death than the general population [14]. Therefore, it is imperative to investigate the biological mechanisms underlying mental health issues in middle-aged and older adults, particularly in the context of social isolation.

The gut microbiota is closely linked to depression. Changes in the diversity and composition of the gut microbial ecosystem have been associated with major depression [15,16]. Patients with depression exhibit elevated levels of Bacteroidetes, Proteobacteria, Actinomycetes, Faecalibacterium, Ruminococcus, Lactobacillus, and Bifidobacterium, and decreased levels of Firmicutes [17]. In germ-free mice colonized with feces from depressed patients, depression-like behavior was observed [18]. The gut microbiota produces a variety of metabolites, including SCFAs and tryptophan, which may alleviate depressive symptoms [19,20]. The gut microbiota regulates tryptophan metabolism by acting on the vagus nerve [21]. However, the impact of chronic social isolation on the gut microbiota remains largely unknown. In the study, 55-week-old mice were used for chronic social isolation treatment to simulate an elderly population living alone. Subsequently, the diversity and composition changes of the gut microbiota were analyzed.

## 2 Materials and methods

### 2.1 Animals

For this study, the experiments were strictly in accordance with protocols approved by the Ethics Committee of Hainan Medical College for Animal Care and Use. The animals were monitored twice per week, and none of the animals showed severe illness, died, or appeared moribund during any of the experiments. All mice were purchased from Changsha Tianqin Biotechnology Co., Ltd, China. The mice were housed in an environment with a temperature of 22 ± 1 °C, relative humidity of 50 ± 5 %, and a light cycle of 12 h of light/12 h of dark. Mice had free access to standard mouse chow, which was composed of 25 % wheat flour, 20 % corn flour, 15 % sorghum flour, 22 % soybean flour, 18 % bran, 4 % fish meal, 2 % bone meal, 1 % yeast meal, 3 % bone meal, 1 % cod liver oil, and 1 % edible salt (Guangdong Medical Laboratory Animal Center) and water. Corn cob bedding (Guangdong Medical Laboratory Animal Center) was placed in the rearing cages. Body weight and health status were monitored weekly.

As depression is an inflammatory disease, Balb/c mice are frequently used in immunological research. A total of 14 male BALB/c mice (55 weeks old) were randomized into 2 groups, with 7 animals in each group. There were 2–3 mice per cage (ZK-M1 mouse cage, material: polypropylene PP/PC material, specification: L*W*H: 290*178*160 (unit: mm), weight: 285 g) in the control group. One mouse was housed per cage in the CSI group. All mice were fed and watered normally during the treatment period.

In the 12th week, the mice were euthanized by chloral hydrate anesthesia. Blood and intestinal contents were taken for further analysis. Blood samples from the hepatic portal vein were collected in tubes containing EDTA anticoagulant, were centrifuged at low speed (1000×*g*, 4 °C) for 15 min, and the plasma was separated and quickly placed in liquid nitrogen and then frozen at −80 °C for subsequent biochemical analysis. The intestinal contents were taken from the mouse colon, separated, quickly placed in liquid nitrogen and transferred to a −80 °C freezer after 10 min for subsequent studies.

### 2.2 Animal behavior tests

Depression-like behavior detection is commonly conducted through the use of three commonly employed tests, namely Tail suspension test (TST), Forced swim test (FST), and Open field experiment [22]. These tests were typically scheduled during the evening, when the mice were in their active period. For TST, the mice were hung upside down by their tails, and an analysis system automatically measures the immobility time (within 5 min). For FST, the mice were forced to swim once for 5 min in a 4-L Pyrex glass beaker containing 3 L of water (24 ± 1 °C). The first immobility time and the total immobility time within 5 min of the test were analyzed. All cylinders were emptied and cleaned before starting a new set of experiments. For open field experiment, the mice were allowed to move freely in a wide experimental chamber. For data analysis, animal behavior was analyzed using the SMART Animal Behavior Video Tracking software (SMART V3.0, Panlab, Spain).

### 2.3 Cytokine analysis

The Th1 (TNF-α and IL-1) and Th2 (IL-4 and IL-6) cytokine analyses were performed using ELISA kits (TNF-α: F0121-A; IL-1β: F2039-A; IL-4: F2165-A; and IL-6: F2163-A) from Shanghai Fanke Industrial Co., Ltd., strictly according to the instructions. Briefly, 50 μL of standards of different concentrations was added to each standard well, followed by the addition of 10 μL of plasma samples was added to the wells to be tested. Then, 100 μL of enzyme labeling reagent was added to each well l, which was incubated for 60 min at 37 °C. The wells were gently washed and left to stand for 30 s before the washing solution was discarded, and this step was repeated 5 times. The colorant was then added to each well, and the color was developed at 37 °C for 15 min. Fifty microliters of termination solution was added to terminate the reaction. The concentration of each cytokine was measured by analyzing the absorbance at a 450-nm light wavelength using the detection limit of the IL-1 assay kit of 3.5 pg/mL, the TNF-α assay kit of 3 pg/mL, the IL-4 assay kit of 10 pg/mL, and the IL-6 assay kit of 3 pg/mL. Additionally, each kit could not react with other cytokines.

### 2.4 Microbiota analysis by 16S rRNA gene sequencing

Total fecal DNA was extracted by a QIAamp DNA Stool Minikit (Qiagen, Hilden, Germany) according to the manufacturer's instructions. First, the MiSeq library was constructed, and the official Illumina adapter sequence was added to the outer end of the target region by PCR. Then, one end of the DNA fragment was base-complementary paired to the primer and fixed on the chip, and MiSeq sequencing was started.

The sequencing was conducted on the Illumina MiSeq PE300 platform (Shanghai Meiji Biomedical Technology Co., Ltd., China), with an average read length of approximately 422 bases. Briefly, the hypervariable V3–V4 region of the microbial 16S rRNA gene was amplified via PCR using the primers 338F (5′-ACTCCTACGGGAGGCAGCA-3′) and 806R (5′-ACTCCTACGGGAGGCAGCA-3′). The resulting PCR product was recovered and purified using an AxyPrep DNA Gel Extraction Kit (Axygen Biosciences, United States) and eluted with Tris-HCl. The purified product was detected via 2 % agarose electrophoresis and quantified using QuantiFluor™-ST (Promega, United States).

The operational taxonomic unit (OTU) clustering and subsampling were performed using the UPARSE (version 7.1) software, which employed a 97 % similarity threshold for clustering sequences [23]. Additionally, chimeras were removed using the UCHIME software [24]. The visualization of the results was performed using Mothur. The Venn diagram, community histogram/heatmap, rarefaction curve, and alpha-diversity indices were analyzed using Mothur. The rank-abundance curve graph was generated using the R language tool. The sample hierarchical clustering and principal coordinates analysis (PCA) were performed to estimate the beta diversity of the community, and the graphics were generated using R language. Each sequence was annotated for species classification by comparing it to the Silva database (SSU123) using the RDP classifier, with the alignment threshold set to 70 %. The UniFrac Server was used to evaluate the community comparison, followed by a Wilcoxon rank sum-test (P < 0.05).

In addition, linear discriminant effect size (LEfSe) analysis was used to find features that were differentially represented between the groups: a nonparametric factorial Kruskal‒Wallis (KW) sum-rank test was used to detect features with significant abundance differences and to find taxa that differed significantly from abundance, followed by a linear discriminant analysis (LDA, threshold 2), with a multigroup comparison strategy of all-against-all (more strict), to estimate the magnitude of the effect of abundance of each component (species) on the differential effect [60].

### 2.5 Metabolome analysis

A 50-mg sample of the intestinal contents was subjected to metabolite extraction with 400 μL of extraction solution (methanol:water = 4:1 (v:v)). The samples were left at −20 °C for 30 min and then centrifuged for 15 min (4 °C, 13,000×*g*), and the supernatant was collected. An equal volume of metabolites from all samples was taken and mixed to prepare quality control (QC) samples.

This study used non-traditional metabolomics to identify distinctive metabolites. Briefly, an ultra-performance liquid chromatography-tandem time-of-flight mass spectrometry system (triple TOFTM5600+, AB Sciex, USA) was utilized for LC-MS analysis, and an HSS T3 column (100 mm × 2.1 mm i.d., 1.8 μm) was employed. A 10-μL sample was separated by a chromatographic column and subjected to mass spectrometry detection. Mobile phase A was 0.1 % formic acid in water, and mobile phase B was 0.1 % formic acid in acetonitrile/isopropanol/water (47.5/47.5/5, v/v/v). The separation gradient was as follows: 0–0.5 min, mobile phase B maintained at 0 %; 0.5–2.5 min, mobile phase B increased from 0 % to 25 %; 0.5–2.5 min, mobile phase B increased from 5 % to 25 %; 2.5–9 min, mobile phase B increased from 25 % to 100 %; 9–13 min, mobile phase B maintained 100 %; 13.0–13.1 min, mobile phase B decreased from 100 % to 0 %; 13.1–16 min, mobile phase B maintained at 0 %. The flow rate was 0.40 mL/min, and the column temperature was 40 °C. The sample mass spectrometry signal acquisition mass scan range (*m*/*z*) was 50–1000, the positive mode ion spray voltage was 5000 V, the negative mode ion spray voltage was −4000 V, the declustering voltage was 80 V, the spray gas was 50 psi, the auxiliary heating gas was 50 psi, the curtain gas was 30 psi, the ion source heating temperature was 550 °C, and the cyclic collision energy was 20–60 V.

The LC/MS raw data were imported into the metabolomics processing software Progenesis QI (Waters Corporation, Milford, USA) for baseline filtering, peak identification, integration, retention time correction, and peak alignment, resulting in a data matrix of retention time, mass-to-charge ratio, and peak intensity. The metabolite information was obtained by matching the MS and MS/MS mass spectrometry information with the metabolic public databases HMDB (http://www.hmdb.ca/) and Metlin (https://metlin.scripps.edu/) and the Majorbio library.

The data obtained by matching were uploaded to Majorbio's BioCloud platform (https://cloud.majorbio.com) for analysis. To analyze the raw metabolome data better, a series of preprocessing steps of the raw data was performed to filter the data according to the proportion of missing values within the sample or subgroups, removing peaks with more than 80 % missing values in the sample. The response intensities of the sample mass spectrometry peaks were normalized using the sum normalization method. Additionally, variables with a relative standard deviation (RSD) > 30 % for QC samples were removed and log10 logarithmized.

The analysis of metabolite differences was performed by principal component analysis (PCA) and orthogonal least squares discriminant analysis (OPLS-DA) using the R software package ROPLS (Version 1.6.2), and the stability of the model was assessed using 7-cycle interactive validation. The selection of differentially expressed metabolites was based on the variable weight values (VIP) and Student's *t*-test p values obtained from the OPLS-DA model, and metabolites with VIP>1 and p < 0.05 were identified as differentially expressed metabolites.

### 2.6 Statistical analysis

Statistical analysis of physiological and biochemical data was conducted using GraphPad Prism version 7.0 (GraphPad Software, San Diego, CA). The Student-T test was used to determine significant mean differences. Data were expressed as mean ± SE. P values less than 0.05 were considered statistically significant.

## 3 Result

### 3.1 Chronic social isolation (CSI) leads to depression-like behavior in mice

**Fig. 1:**
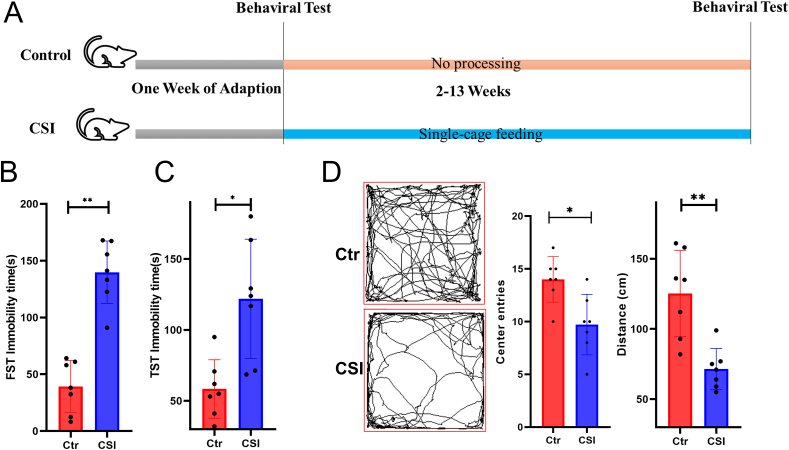
**Chronic social isolation leads to the development of depression.** A. Mice underwent 12 weeks of single-cage housing. Mouse animal behavior was tested. B. FST and C. TST immobility time within 5 min, and D. percentage of time engaged in slow activity.

Prior to the experiment and after 12 weeks of being housed individually, the mice underwent behavioral testing (Fig. 1A, Fig. S1). The results of the forced swim test showed that the immobility time of the CSI mice was significantly increased compared with that of the Ctr mice (P < 0.05) (Fig. 1B). Similarly, the tail suspension test revealed a significant increase in immobility time for the CSI group (P < 0.05) (Fig. 1C). In the open field test, the CSI group of mice had shorter distances of movement and fewer crossings through the central zone compared to the control group (Fig. 1D).

### 3.2 Changes in the gut microbiota composition in chronic social isolation mice

The gut microbiota of mice was analyzed using high-throughput sequencing of 16S rRNA. The results from the Shannon, Simpson, Ace, and Chao indices showed no significant changes in the alpha diversity in the CSI group compared to the control (Ctr) group (Fig. S2A). However, PCA analysis and PLS-DA analysis revealed significant differences in the beta diversity of the gut microbiota between the Ctr and CSI groups of mice (Figs. S2B and C).

**Fig. 2:**
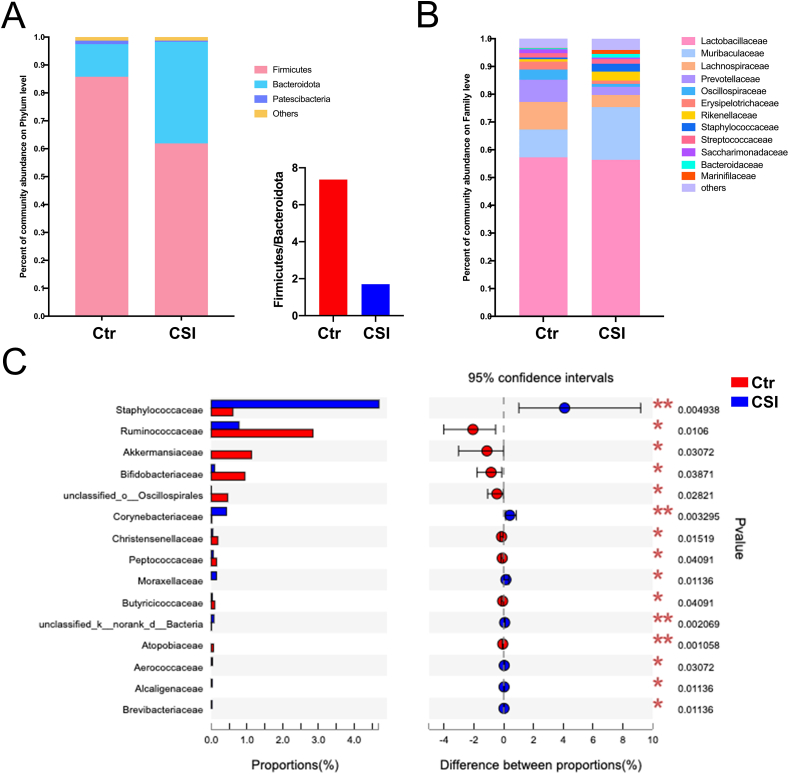
**Changes in the composition of the gut microbiota due to chronic social isolation.** A. Changes in the composition at the phylum level, especially a decrease in the Firmicutes/Bacteroidetes ratio. B. Changes in the composition at the family level. C. Bacteria with significant changes at the family level.

At the phylum level, Firmicutes, Bacteroidetes, and Patescibateria were the dominant gut microbiota. Chronic social isolation led to a decrease in the abundance of Firmicutes and an increase in Bacteroidetes in the CSI group compared to the Ctr group (Fig. 2A). Additionally, the Firmicutes-to-Bacteroidetes ratio was decreased in the CSI group.

At the family level, the Wilcoxon rank sum test results showed that the abundances of Staphylococcaceae, Corynebacteriaceae, Moraxellaceae, Aerococcaceae, Alcaligenaceae, and Brevibacteriaceae were significantly increased, while the abundances of Ruminococcaceae, Akkermansiaceae, Christensenellaceae, Peptococcaceae, Butyricicoccaceae, and Atopobiaceae were significantly decreased in the CSI group compared to the Ctr group (Fig. 2B and C).

### 3.3 Characteristic bacteria in the gut microbiota of chronic social isolation mice

**Fig. 3:**
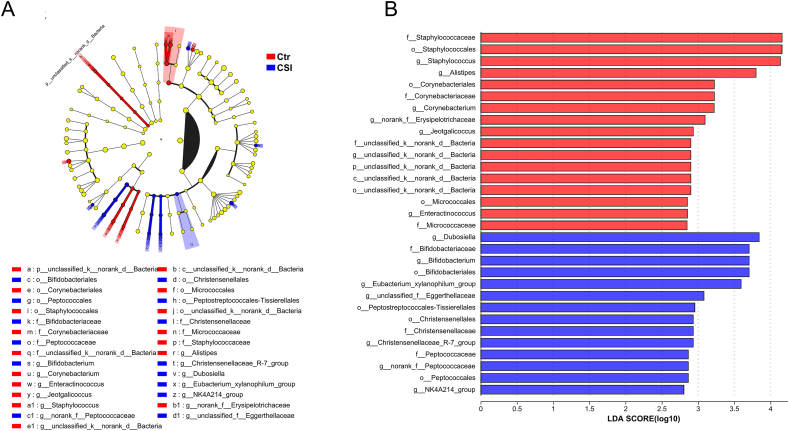
**LEfSe analysis of the characteristic bacteria associated with chronic social isolation.** A. LEFSe multilevel species hierarchical tree. B. LDA discriminant results.

To understand the gut microbiota associated with chronic social isolation, LEfSe was used to analyze the characteristic bacteria (Fig. 3A and B). At the genus level, *Staphylococcus*, *Alistipes*, *Corynebacterium*, *Jeotgallcoccus*, and *Enteractinococcus* (linear discriminant analysis (LDA) scores >2 and P < 0.05) were significantly different between the CSI group and the control group.

### 3.4 Changes in metabolites of the gut microbiota in chronic social isolation mice

**Fig. 4:**
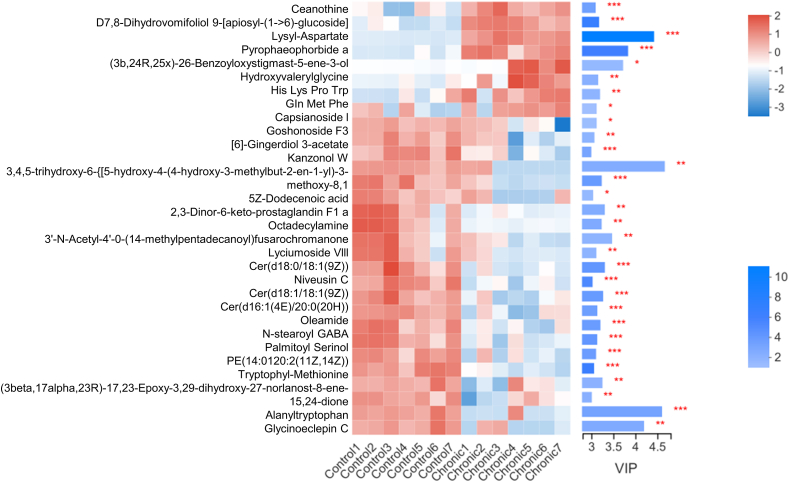
**Chronic social isolation leads to changes in the metabolites of the gut microbiota.** Among the 30 metabolites, 8 had a higher abundance, and 22 had a lower abundance in the chronic social isolation group.

Since changes in the composition of the gut microbiota can affect its metabolites, the effects of chronic isolation on the levels mouse gut microbiota metabolites were analyzed by clustering and VIP (Fig. 4). In the CSI group, 30 metabolites were significantly changed, of which 8 were significantly increased and 22 were significantly decreased. Metabolites that were significantly elevated included lysyl-aspartate, pyrophophorbide a, hydroxyvalerylglycine, His_Lys_Pro_Trp, and Gln_Met_Phe. Metabolites that were significantly decreased included capsianoside I, goshonoside F3, [6]-gingerdiol 3-acetate, kanzonol W, lyciumoside VIII, niveusin C, tryptophyl-methionine, oleamide, N-stearoyl GABA, and palmitoyl serinol.

**Fig. 5:**
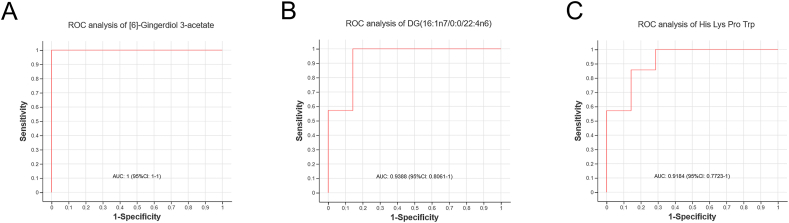
**ROC analysis of the gut microbiota metabolites.** A. [6]-Gingerdiol 3-acetate, b. DG (16:1n7/0:0/22:4n6), and c. His_Lys_Pro_Trp.

Based on metabolomics assays, ROC curves were used to analyze the intestinal metabolites in chronic social isolation mice, and three metabolites were considered to be highly sensitive (Fig. 5A–C). The areas under the curve of [6]-gingerdiol 3-acetate, DG (16:1n7/0:0/22:4n6), and His_Lys_Pro_Trp were 1 (95 % CI 1-1), 0.9388 (95 % CI 0.6061–1) and 0.9184 (95 % CI 0.7723–1), respectively.

## 4 Discussion

This study was conducted on 55 week old aged mice and for a duration of 12 weeks. In the animal behavioral tests conducted, the immobility time of the mice increased significantly in both TST and FST experiments, as well as slower movement speed and fewer entries into the intermediate area in the open-field experiment. These results are consistent with previous reports that chronic social isolation can result in the development of depressive symptoms [25,26].

This study focuses on the alterations in the gut microbiota and their metabolites in middle-aged mice subjected to chronic social isolation. Although the microbial OTU count and alpha diversity remained unchanged by prolonged solitude, the application of principal component analysis (PCA) and partial least squares discriminant analysis (PLS-DA) revealed significant changes in the beta diversity of the gut microbiota in the chronic social isolation (CSI) group (Fig. S2). Additionally, the Firmicutes-to-Bacteroidetes ratio exhibited a significant decrease in the CSI group. Notably, this investigation highlighted a significant increase in disease-associated bacteria, including Staphylococcaceae, Corynebacteriaceae, Euphorbiaceae, Aerococcaceae, and Calcitonellaceae, while probiotic bacteria, such as Ruminococcaceae, Akkermansiaceae, and Christensenellaceae, were significantly decreased in the CSI group.

The present study demonstrates that the ratio of Firmicutes to Bacteroidetes in the intestinal microbiota was decreased in mice subjected to chronic social isolation. Firmicutes and Bacteroidetes are the two dominant phyla in the gut microbiota, accounting for more than 90 % of the total bacterial population. These phyla are relatively stable and less susceptible to acute disturbances [27]. The ratio of Firmicutes to Bacteroidetes has been shown to reflect the composition of the human gut microbiota and is associated with various diseases [28], including diabetes, obesity, and Alzheimer's disease [29]. Furthermore, the Firmicutes-to-Bacteroidetes ratio has been found to be decreased in the gut microbiota of patients with major depressive disorder (MDD) [[30], [31], [32], [33]] and may serve as an important indicator for distinguishing depressed patients from healthy individuals [34]. Therefore, our findings suggest that the Firmicutes-to-Bacteroidetes ratio could be a useful indicator for depression induced by chronic social isolation.

Our study demonstrated that the abundances of Staphylococcaceae, Corynebacteriaceae, Moraxellaceae, Aerococcaceae, and Alcaligenaceae were significantly increased in the chronic social isolation (CSI) group. Staphylococcus is a crucial pathogen responsible for hospital-acquired infections, and the extensive use of antibiotics in clinical practice has led to the emergence of multidrug-resistant strains, which pose a significant challenge in the treatment of clinical infectious diseases [35]. Corynebacteriaceae is a conditionally pathogenic bacterium that is associated with upper respiratory tract infections and lung infections [36]. The increased abundance of Moraxellaceae was positively correlated with pneumonia [37]. Aerococcaceae is associated with a variety of infectious diseases [38,39]. Alcaligenaceae is believed to be associated with focal infections and bacteremia [40]. Therefore, our findings suggest that chronic social isolation could promote the proliferation of disease-associated bacteria.

Moreover, chronic social isolation in mice was associated with a decrease in the abundance of Ruminococcaceae, Akkermansiaceae, and Christensenellaceae. Recent studies have reported a decrease in the abundance of these bacterial families in patients with depression [41]. Ruminococcaceae possesses anti-inflammatory properties and can prevent abnormal microglial phagocytosis, thereby reducing depressive-like behavior [42,43]. Decreased Christensenellaceae has been associated with affective disorders, as well as increased oxidative stress and systemic low-grade inflammation [44,45]. Additionally, these bacterial families have been linked to the production of SCFAs [46,47], which can cross the blood-brain barrier, regulate microglial immune function, and reduce neuroinflammation [48]. A reduction in SCFA-producing bacteria has been observed in various neurodegenerative diseases, including depression and Parkinson's disease [48]. Therefore, our study demonstrates that chronic social isolation leads to a reduction in SCFA-producing bacteria.

This study demonstrates that chronic social isolation (CSI) can induce changes in the metabolites of the gut microbiota, which in turn have effects on the nervous system. Several studies have demonstrated that oleamide can alleviate depressive symptoms in chronically mildly stressed rats [49]. Tryptophan can pass through the blood-brain barrier to replenish brain consumption [50]. When indoleamine 2,3-dioxygenase 1 is overactivated, tryptophan in brain tissue is used to generate kynurenine, which reduces 5-HT synthesis, an important cause of depression [51]. Furthermore, the gut microbes can directly convert tryptophan into a number of molecules, including aryl hydrocarbon receptor (AhR) ligands, maintenance of barrier integrity [52]. In this study, the contents of oleamide and tryptophyl-methionine decreased in the intestinal contents of CSI mice. Therefore, this study demonstrates that chronic social isolation can induce changes in the metabolites of the gut microbiota, which in turn have effects on the nervous system.

Systemic inflammation is a significant cause of mental disorders [53]. Excessive levels of the systemic inflammatory biomarker IL-6 have been associated with loneliness [54,55]. Prolonged high levels of IL-6 have also been linked to the development of depression [56]. Additionally, elevated levels of proinflammatory cytokines IL-1 and IL-6 have been observed in the peripheral blood of MDD patients [57]. In a study conducted by DiStefano et al., transplantation of feces from depressed patients to normal mice resulted in elevated levels of the IL-1 [58]. These findings suggest that chronic social isolation may trigger inflammatory responses, which may contribute to the development of depression.

### 4.1 Plasma cytokines are significantly increased in chronic social isolation mice

**Fig. 6:**
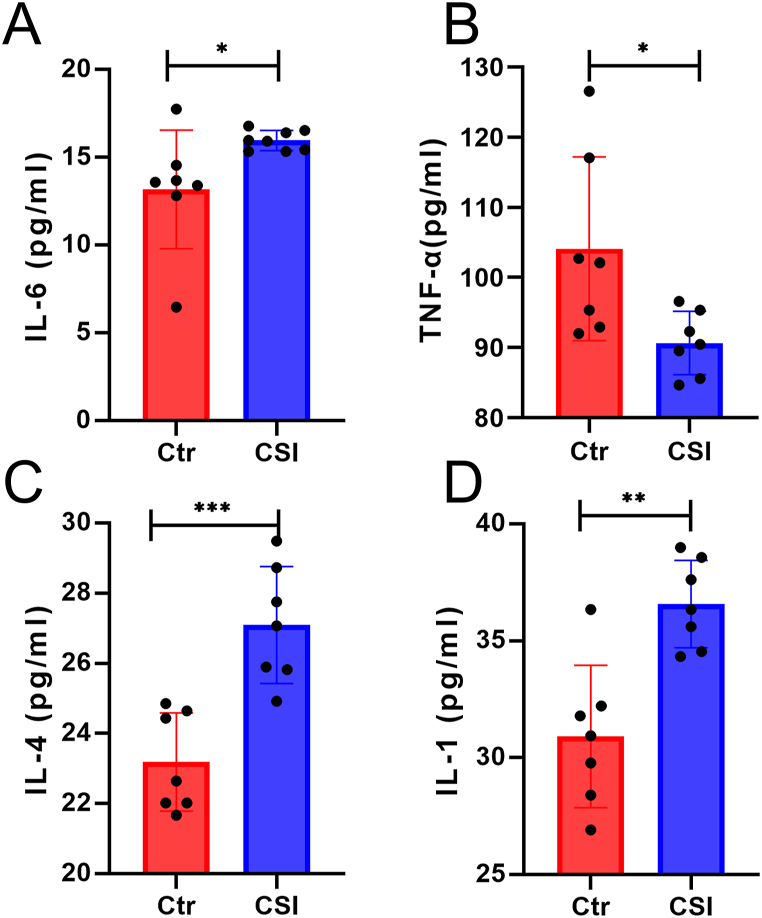
**Chronic social isolation leads to changes in the levels of some inflammatory factors.** A. IL-1, B. TNF-α, C. IL-4, D. IL-6.

In the CSI group, the level of the Th1 cytokine IL-1β showed a significant increase, while that of TNF-α exhibited a significant decrease (Fig. 6A and B). Additionally, the Th2 cytokines IL-4 and IL-6 were significantly elevated in the CSI group (Fig. 6C and D).

## 5 Conclusion

Our study demonstrates that chronic social isolation induces depression-like behavior in mice by altering their gut microbiota. The Firmicutes-to-Bacteroidetes ratio is affected by chronic social isolation, resulting in a reduction in the abundance of SCFA-producing bacteria and an increase in proinflammatory and disease-related bacteria. Additionally, chronic social isolation alters the gut microbiota metabolites, particularly by inhibiting the production of neuro-related metabolites such as oleamide and tryptophan. Furthermore, the levels of proinflammatory cytokines were significantly increased. These findings suggest that chronic social isolation induces changes in the microbial composition and function, leading to chronic inflammation and mental health impairment. However, the effects of chronic social isolation on the gut microbiota might vary with the age of the mice. Further research is needed to analyze the changes in the gut microbiota in different ages of mice.

## Ethics approval and consent to participate

The use of mice and all experimental procedures were reviewed and approved by the Ethics Committee of Hainan Medical University (HY-2022-04-1801).

## Consent for publication (MISSING)

Not applicable.

## Data availability statement

All datasets generated and analyzed during the current study were uploaded with the manuscript as additional files.

## Funding

This study was funded by the 10.13039/501100004761Natural Science Foundation of Hainan (821RC561) (Guankui Du).

## CRediT authorship contribution statement

**Linwei Ding:** Writing – original draft. **Jiaqi Liu:** Data curation. **Yunjia Yang:** Data curation. **Zeying Cui:** Data curation. **Guankui Du:** Writing – review & editing, Project administration, Methodology, Funding acquisition, Formal analysis, Data curation.

## Declaration of competing interest

The authors declare that they have no known competing financial interests or personal relationships that could have appeared to influence the work reported in this paper.
