# Supplementary material for: Chronically socially isolated mice exhibit depressive-like behavior regulated by the gut microbiota
Source: Heliyon. 2024 Apr 18;10(8):e29791. doi: 10.1016/j.heliyon.2024.e29791 (PMC11046198; doi:10.1016/j.heliyon.2024.e29791)
Supplement: Multimedia component 1 [file mmc1.docx]

**Supplementary Figure legends**


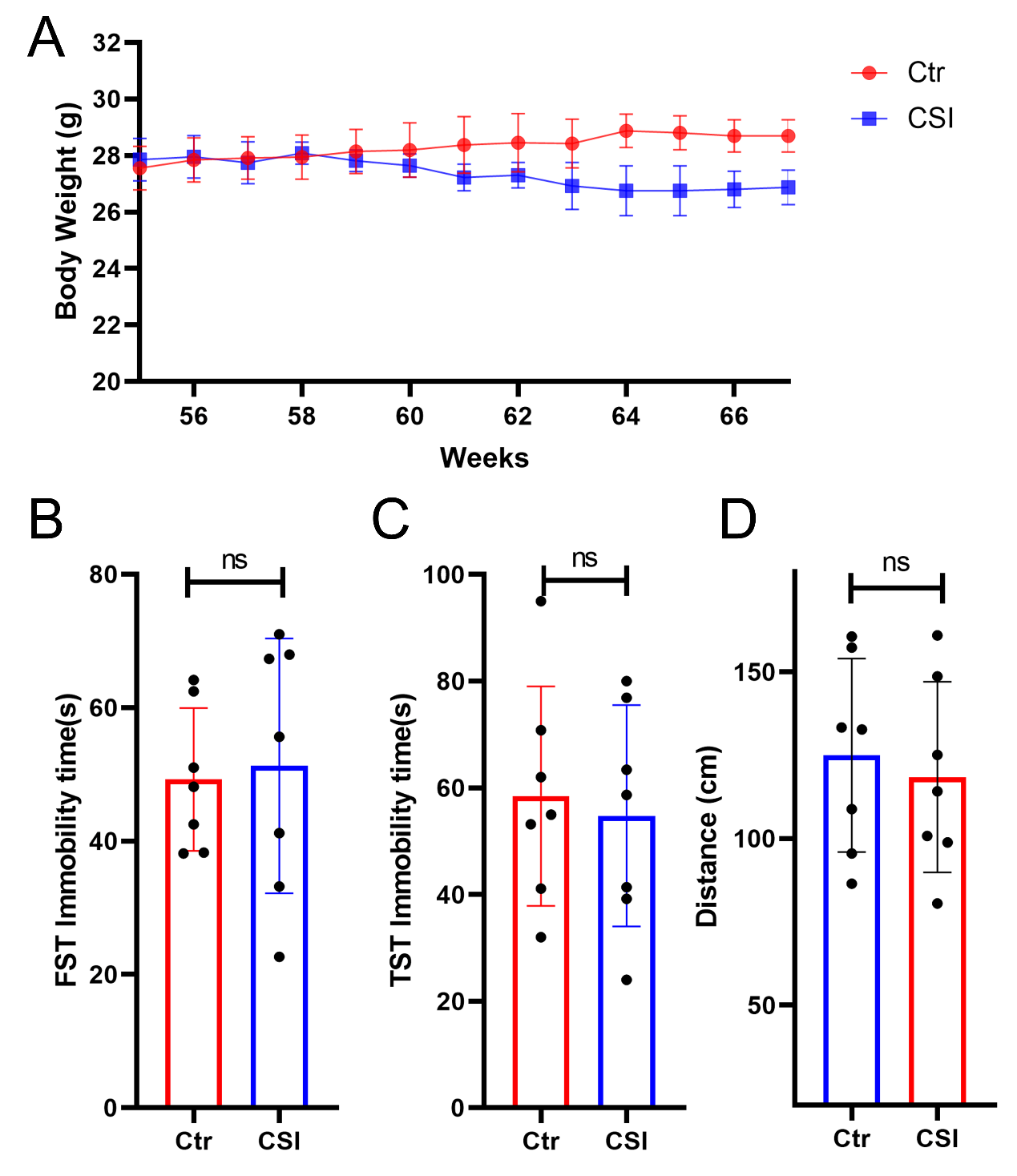


**Figure S1. Behavioral tests of animals before chronic social isolation and changes in body weight of mice.**


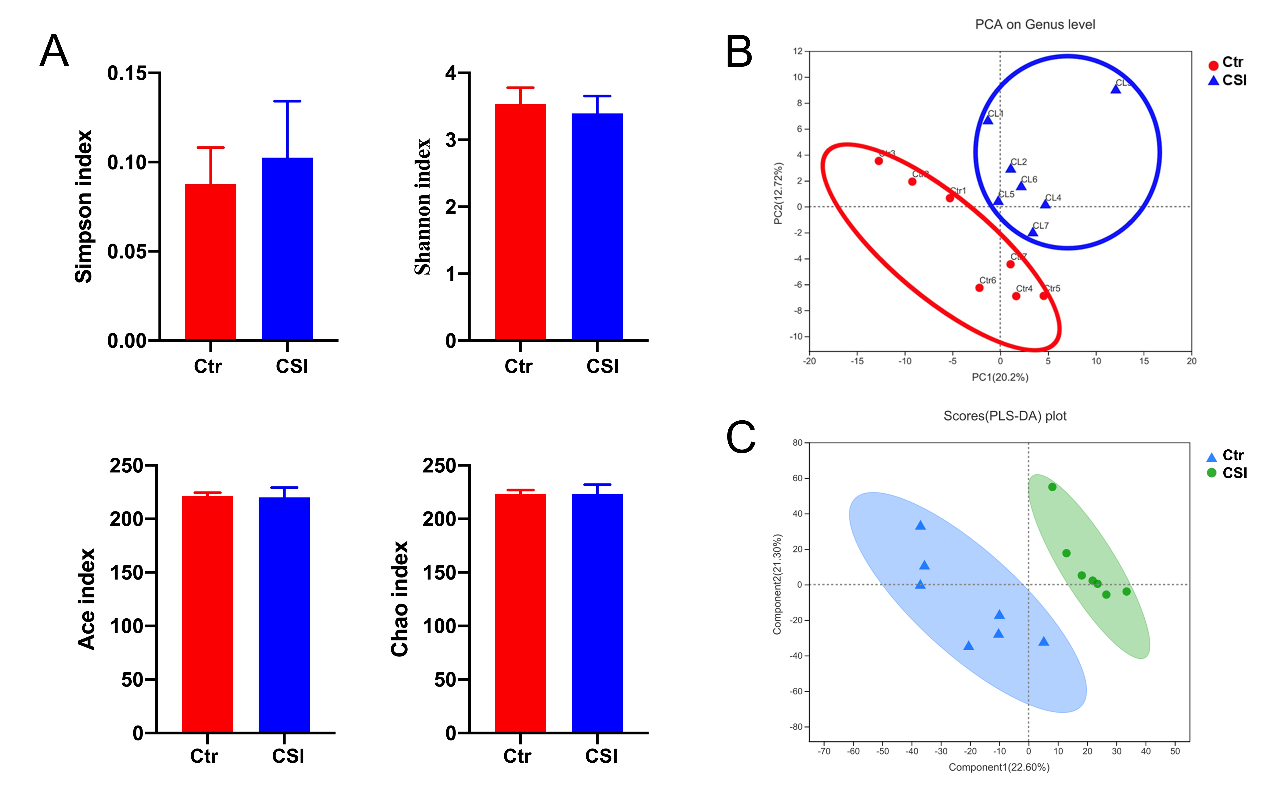


**Figure S2. Diversity analysis of the mouse gut microbiota.** A. Alpha diversity analysis. Beta diversity analysis. B. PCA and C. PLS-DA analysis.
